# Aging, Plasminogen Activator Inhibitor 1, Brain Cell Senescence, and Alzheimer’s Disease

**DOI:** 10.14336/AD.2022.1220

**Published:** 2023-04-01

**Authors:** Chun-Sun Jiang, Tapasi Rana, Lee-Way Jin, Susan A Farr, John E Morley, Hongwei Qin, Gang Liu, Rui-Ming Liu

**Affiliations:** ^1^Department of Medicine, University of Alabama at Birmingham (UAB), Birmingham, AL, USA.; ^1^Department of Pathology and Laboratory Medicine, University of California, Davis, CA, USA.; ^3^Division of Geriatric Medicine, School of Medicine, Saint Louis University, St. Louis, MO, USA.; ^4^Research and Development, Veterans Affairs Medical Center, St. Louis Missouri, MO, USA.; ^5^Department of Cell, Developmental and Integrative Biology, UAB, Birmingham, AL, USA.

**Keywords:** Aging, PAI-1, cellular senescence, Alzheimer’s disease;

## Abstract

The etiology for late-onset Alzheimer’s disease (LOAD), which accounts for >95% of Alzheimer’s disease (AD) cases, is unknown. Emerging evidence suggests that cellular senescence contributes importantly to AD pathophysiology, although the mechanisms underlying brain cell senescence and by which senescent cells promote neuro-pathophysiology remain unclear. In this study we show for the first time that the expression of plasminogen activator inhibitor 1 (PAI-1), a serine protease inhibitor, is increased, in correlation with the increased expression of cell cycle repressors p53 and p21, in the hippocampus/cortex of senescence accelerated mouse prone 8 (SAMP8) mice and LOAD patients. Double immunostaining results show that astrocytes in the brain of LOAD patients and SAMP8 mice express higher levels of senescent markers and PAI-1, compared to astrocytes in the corresponding controls. In vitro studies further show that overexpression of PAI-1 alone, intracellularly or extracellularly, induced senescence, whereas inhibition or silencing PAI-1 attenuated H_2_O_2_-induced senescence, in primary mouse and human astrocytes. Treatment with the conditional medium (CM) from senescent astrocytes induced neuron apoptosis. Importantly, the PAI-1 deficient CM from senescent astrocytes that overexpress a secretion deficient PAI-1 (sdPAI-1) has significantly reduced effect on neurons, compared to the PAI-1 containing CM from senescent astrocytes overexpressing wild type PAI-1 (wtPAI-1), although sdPAI-1 and wtPAI-1 induce similar degree of astrocyte senescence. Together, our results suggest that increased PAI-1, intracellularly or extracellularly, may contribute to brain cell senescence in LOAD and that senescent astrocytes can induce neuron apoptosis through secreting pathologically active molecules, including PAI-1.

## INTRODUCTION

Aging is the greatest risk factor for late-onset Alzheimer’s disease (LOAD), which accounts for >95% of AD cases. The mechanism(s) underlying the aging-related susceptibility to LOAD, however, is unknown. Cellular senescence, which occurs in both proliferative cells and post-mitotic cells, represents a state of the permanent arrest of cell growth. Besides loss of normal functions and regeneration capacity, senescent cells secrete an array of pathogenically active molecules, termed senescence associated secretory phenotype (SASP), which adversely affect the functions of secreting cells and surrounding tissues/cells. Emerging evidence suggests that cellular senescence contributes importantly to the patho-physiology of aging and aging-related diseases [1-3], including AD [4-7]. Senescent cells have been detected in the brain of AD patients [5-8] and AD model mice [4-6]. Removal of senescent cells pharmacologically or genetically alleviated brain Aβ accumulation and tauopathy, two pathological features of AD, and improved memory in AD model mice [4-6], suggesting that cell senescence contributes importantly to the neuro-pathophysiology of AD. Nonetheless, what triggers brain cell senescence during aging and in AD, and how cell senescence leads to the pathophysiology of AD remain largely unknown. Finding answers to these questions will not only help understanding the mechanisms underlying AD but may also lead to identification of novel therapeutic targets for the treatment of this devastating disease.

Plasminogen activator inhibitor 1 (PAI-1), a primary inhibitor of tissue-type and urokinase-type plasminogen activators (tPA and uPA), plays a central role in hemostasis. Studies from this lab and others have shown that PAI-1 expression increases with age in the plasma [9, 10] as well as in the brain of AD patients [11-14] and AD model mice [4, 11, 15, 16]. Inhibition of PAI-1 activity or ablation of PAI-1 reduced brain Aβ load and improved memory in familial AD model mice [11, 15-17], suggesting an essential role of PAI-1 in the pathophysiology of familial AD. Whether and how increased PAI-1 contributes to the neuropathophysiology in LOAD, the etiology, and the mechanisms of which is clearly different from FAD, however, is unknown and warrant further investigation [18-20].

In this study, we tested a hypothesis that aging-related increase in PAI-1 contributes to brain cell senescence and thus the neuropathophysiology during aging and in LOAD, using senescence accelerated murine prone 8 (SAMP8) mice, which harbor many behavioral and histopathological signatures of AD [21-27], and brain tissue samples from LOAD patients and age-matched healthy controls. Pharmacological and genetic approaches were used to modulate PAI-1 activity and expression to define the role of PAI-1 in astrocyte senescence as well as the contribution of astrocyte SASP in neuron apoptosis in vitro. Our results support the notion that increased PAI-1 plays a critical role in brain cells, especially astrocyte, senescence and senescent astrocytes secrete pathologically active molecules, including PAI-1, which promote neuron apoptosis.

## MATERIALS AND METHODS

### Human samples

High-quality human brain samples from the dorsolateral prefrontal cortex were obtained at autopsy from the longitudinal cohorts of the Alzheimer's Disease Research Center (ADRC, P30 AG072972) at University of California at Davis. The subjects had been well characterized by clinical, neuroimaging, neuro-psychiatric, and post-mortem neuropathological analyses as: 1) those diagnosed with LOAD with high AD neuropathological changes (Braak stage V or VI, CERAD score 2 or 3, Thal phase 4 or 5) according to the current NIA-AA guidelines [28, 29]; 2) Age-matched healthy individuals with minimal cognitive complaints and low AD neuropathological change (Braak stage < II, CERAD score 0 or 1, Thal phase <2) (Table 1) as well as no significant vascular, Tau, TDP-43, or α-synuclein pathology. The samples had been de-identified at UCD Center before being delivered to us to protect the privacy of the individuals.

**Table 1:** Characterization of patients and age-matched healthy controls.

NA ID	age	Sex	AD Neuropathological changes	Thal phase	Braak state	Diagnosis
1	90	F	A3, B1, C1	4	II	Low likelihood AD
2	84	F	A0, B1, C0	0	I	Not AD
3	90+	F	A1, B1, C2	2	II	Low likelihood Ad
4	90+	F	A0, B1, C0	0	II	Not AD
5	78	F	A1, B2, C0	1	III	Low likelihood AD
6	87	F	A3, B3, C3	5	VI	High likelihood AD
7	87	F	A3, B3, C3	4	V	High likelihood AD
8	75	F	A3, B3, C2	5	VI	High likelihood AD
9	89	F	A3, B3, C3	5	VI	High likelihood AD
10	86	F	A3, B3, C3	5	VI	High likelihood AD
11	77	F	A3, B3, C3	5	VI	High likelihood AD
12	90+	F	A2, B3, C3	3	VI	Intermediate likelihood AD
13	65	F	A3, B3, C3	5	VI	High likelihood AD
14	87	F	A3, B3, C3	5	VI	High likelihood AD
15	68	F	A3, B3, C3	5	VI	High likelihood AD

### Mice

Senescence accelerated mouse prone 8 (SAMP8) mice have been described in our previous works [30, 31] and CD-1 mice were from Charles River. The 50% SAMP8 mice were re-derived from the SAMP8 and CD-1 mice as previously described [32]. Four and twelve 12-month-old SAMP8 and wild type (CD-1) mice were sacrificed, and the hippocampus and cortex were dissected and fixed in 10% formalin or frozen down immediately in liquid nitrogen. All procedures involving animals were approved by the Institutional Animal Care and Use Committees at the University of Alabama at Birmingham.

### Astrocyte isolation

Primary mouse astrocytes were isolated from cerebral cortex of neonatal C57BL/6J wild type mice and PAI-1 deficient mice (The Jackson Laboratory, Bar Harbor, ME) as we have described previously [33]. Briefly, the mouse brains were dissected, and the meninges were carefully removed. A mechanical dissociation of the cortices was performed by passing the tissue through a 1 ml sterile pipette. The homogenate was filtered through 100 μm cell strainer and then centrifuged at 800 x g for 5 min at 4°C. The collected cell mixture was cultured in T75 flasks (one brain per flask) in high glucose DMEM supplemented with 10% FBS, non-essential amino acids (1X), penicillin/streptomycin (1X), 16 mM HEPES and 2 mM glutamine. After 2-week culture, the flasks were shaking on an orbital shaker at ~250 rpm for 5 hours to remove the microglial cells. The remaining astrocytes were trypsinized for further experiments.

### Generation of secretion deficient PAI-1 (sdPAI-1) expressing lentivirus

Truncated mouse PAI-1 (mPAI-1), which does not have the signal peptides for secretion, was PCR amplified from a wild type PAI-1 expressing lentivirus vector, pCDH-mPAI-1 lentivirus, using forward primer 5'-CT CGGATCCGCCACCATG TTCACTTTACCCCTCCG AG-3' and reverse primer 5'-CGGCGGCCGCTCAAG GCTCCATCACTTGGCCC-3'. The PCR product was digested by BamHI and NotI and cloned into empty pCDH-CMV-MCS-EF1-copGFP lentivirus vector. The correctly truncated mPAI-1 sequence was confirmed by DNA sequencing. The lentivirus was packaged in 293T cells and transduced into target cells as company’s instruction (System Biosciences, Palo Alto, CA USA).

### Cell culture and treatment

Primary human astrocytes (ScienCell Research Laboratories, Catlog No #1800, Carlsbad, CA USA), cultured with Astrocyte Medium (Catlog No #1801, Carlsbad, CA USA), were transfected with non-targeted (NT) siRNA or PAI-1 siRNA (Santa Cruz, SC-37007 and SC-36179) for 24 hours and then treated with 200 µM H_2_O_2_ in serum-free medium for 2 days. Alternatively, the cells were infected with control and wild type PAI-1 expressing lentivirus overnight and then treated with 200 μM H_2_O_2_ in the culture medium with no FBS nor virus for 2 days.

Primary mouse astrocytes isolated from wild type and PAI-1 deficient (PAI-1^-/-^) mice were cultured in 50% DMEM and 50% Neuro Basal medium with 10% FBS, 5 μg/ml NAC, 5 ng/ml HBEGF, 100 U/mL penicillin, 100 μg/mL streptomycin, and 50 μg/mL gentamicin, and treated with 200 µM H_2_O_2_ in culture medium without FBS for 2 days. Alternatively, primary mouse astrocytes from PAI-1^-/-^ mice were infected with control lentivirus or lentivirus expressing wild type (wtPAI-1) or secretion deficient PAI-1 (sdPAI-1) for 24hrs. Then, the viruses were removed, and the cells were cultured with fresh medium without virus and serum for 2 days before being harvested.

U87 MG human glioblastoma cell line was obtained from the American Type Culture Collection (Manassas, VA USA) and cultured with DMEM medium supplemented with 10% fetal bovine serum, 100 units/mL penicillin, and 100 μg/mL streptomycin at 5% CO_2_ and 37 °C. U87 cells were transfected with non-target siRNA or PAI-1siRNA as described above and then treated with 200 μM H_2_O_2_ for 2 days. Alternatively, the cells were treated with H_2_O_2_ in the presence or absence of 25 μM TM5275 for 2 days.

Primary mouse cortical neurons were obtained from Thermo Fisher Scientific (Waltham, MA USA) and cultured with Neurobasal Medium supplemented with 0.2mM GlutaMAX-I Supplement, 2% B-27 Supplement, 100 units/mL penicillin, and 100 µg/mL streptomycin at 5% CO_2_ and 37°C. For the treatment of neurons with the conditional medium (CM) from senescent astrocytes, half of the medium in each well was replaced with the CM. The neurons were cultured with the CM for 2 days.

### Immunofluorescence staining

To identify senescent astrocytes in SAMP8 mouse brain, double immunostaining was conducted using formalin-fixed tissue slides with rabbit monoclonal antibody to MacroH2A1.1 (Cell signaling, Cat No 12455, Danvers, MA USA), a cell senescence marker, and mouse monoclonal antibody to glial fibrillary acidic protein (GFAP, Cell signaling, Cat No 3670, Danvers, MA USA), an astrocyte marker, as we have described previously [34]. To reveal PAI-1 and p16 (a senescence marker) positive astrocytes in brain tissues of LOAD patients and healthy controls, mouse monoclonal anti-PAI-1 (Molecular Innovations, Cat No MA-33H1F7) or mouse monoclonal anti-p16 (Santa Cruz Biotech, Cat No SC-1661) and rabbit polyclonal anti-GFAP antibody (Sigma-Aldrich, Inc., Cat No G9269, St. Louis, MO USA) were used. After overnight incubation of the primary antibody at 4°C, the slides were then incubated with fluorescence-conjugated secondary antibodies (Vector Laboratories, Cat No TI-2000, FI-1000, Newark, CA USA) for 1 h. To confirm the specificity of the staining, isotope controls, the normal mouse IgG (Santa Cruz, Cat No SC-2025) and normal rabbit IgG (R&D Systems, Cat No AB-105-C), instead of antibodies to specific proteins, were included in each experiment (no clear staining from these isotope controls was detected, suggesting that the signals are specific). Slides were mounted with Anti fade Mounting Medium with DAPI (Vector Laboratories, Cat No H-1200, Newark, CA USA). Images were taken with a Nikon Andor Clara camera (Nikon). Over 300 astrocytes cells were counted in 6-9 different areas per mouse/human brain tissue; MacroH2A-, p16-, or PAI-1-positive astrocytes were counted, and the results were expressed as percentages of total astrocytes.

**Figure 1.**
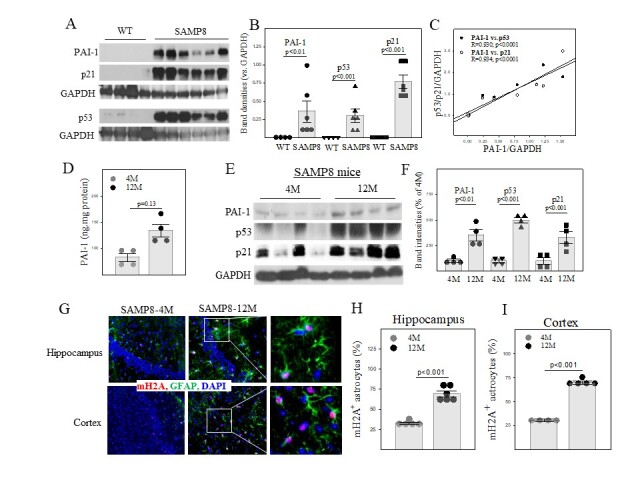
PAI-1 protein level is increased, in correlation with increases of cell cycle repressors, in the hippocampus/cortex of old SAMP8 mice. (A, B) Western analyses and quantification of PAI-1, p53 and p21 proteins in hippocampus/cortex of 12-month-old wild type and SAMP8 mice. The band intensities were normalized by GAPDH bands (n=4-6). (C) Pearson correlation analysis of PAI-1 protein level and p53/p21 protein level (n=10). (D) ELISA of PAI-1 protein in the hippocampus/cortex of young and old SAMP8 mice (n=4-6). (E, F) Western analyses and quantification of proteins of interest in the hippocampus/cortex of 4-month and 12-month-old SAMP8 mice. The results were expressed as percentage of 4M-old mice. (G-I) Immunostaining and quantifications of senescent astrocytes in hippocampus and cortex of 4-month and 12-month-old SAMP8 mice by double immunostaining of mH2A and GFAP (n=4-6). The results were expressed as percentage of total cells.

### Western Blot Analysis

Brain tissues were homogenized in a tissue extraction buffer containing 2% SDS and protease inhibitor (Sigma, St. Louis, MO, USA P8340) and phosphatase inhibitor cocktails (Sigma, P5726). The homogenates were centrifuged at 3000 x g, 4°C, for 10 min and then in 100,000 x g for 60 min. Westerns were conducted with supernatants and the following antibodies: PAI-1 (ASMPAI-GF, ASHPAI-GF, Molecular Innovation, Novi, MI, USA), GFAP (Cat No G9269, Sigma-Aldrich, Inc., St. Louis, MO USA), p53 (SC-6243, Santa Cruz), p21 (SC-397, Santa Cruz, Dallas, TX, USA) and GAPDH (G9545, Sigma). The protein bands were visualized using the ECL detection system (Amersham, Piscataway, NY, USA), semi-quantified by ImageJ software. The results were normalized with GAPDH, a housekeeping gene.

### Measurement of the activity of senescence associated beta-galactosidase (SA-β-gal)

The activity of SA-β-gal in cultured cells was determined using 5-bromo-4-chloro-3-indolyl P3-D-galactoside (X-gal), following the protocol we have described previously [34]. SA-β-gal positive cells (blue color) were counted under microscope and expressed as percentages of total cells.

### Analysis of Apoptotic neurons

Neuron apoptosis was assessed by three techniques: 1) Western analysis of apoptosis markers cleaved caspase 3 and Bax. 2) The activities of Caspase 3/7 and caspase 9: The activities of caspase 3/7 and caspase 9 in the neuron culture medium were assessed using assay kits from Promega (Madison, WI; Caspase-Glo 3/7 Assay) according to the manufacturer’s protocols. 3) TUNEL staining techniques were used to detect apoptotic mouse neurons, using a kit from Sigma (Roche, Sigma 11684795910). Briefly, the cortical neurons (4×10^4^ per well) were plated on 8-chamber slide (Thermo Scientific™ 154534) coated with poly-L-lysine (Gibco A38904-01). After cultured with the CM for 48 hours, the cells were fixed with 4% paraformaldehyde for 15 min at room temperature and then washed with phosphate buffered saline (PBS) 3 times. The cells were permeabilized with Triton® X-100 and incubated with TUNEL staining reaction mixture (Roche, Sigma 11684795910). Fluorescence images were taken at UAB High resolution Image Facility and apoptotic neurons were counted manually in multiple areas for each well. The results are expressed as percentage of apoptotic neurons.

### Statistical analysis

All of the “n” numbers presented in the Figure legends are the numbers of individual person/mouse/treatment, not replication of the same samples. Multiple group data were evaluated by one-way ANOVA while two group data were evaluated by t-test. Normality tests were conducted with Shapiro-Wilk program before group comparison and passed for all of the data, except the data presented in Fig. 4C, D, O and Fig 5H. Following one-way ANOVA, All Pairwise Multiple Comparison (Fisher LSD) was conducted to determine the significance of the difference between any two groups. For the data presented in Fig. 4C, D, O and Fig. 5H, which failed normality tests, the statistical analyses were conducted using Variance on Ranks, followed by Student-Newman-Kauls test. Correlation studies were performed with Pearson Product Moment Correlation program. Statistical significance was determined post-hoc by Tukey’s test.

## RESULTS

### Correlated increases in PAI-1 and cell cycle repressors in the hippocampus/cortex of senescence accelerated mouse prone 8 (SAMP8) mice

SAMP8 mice are well-characterized murine model of accelerated aging, which harbor many behavioral and histopathological signatures of AD, including Aβ deposition, tauopathy [31, 35, 36], and memory deficits [26, 31, 35-37], and have been widely used to study the aging-related neuropathophysiology and screen candidate drugs for AD [26, 31, 35-37]. Despite intensive characterization, the genes responsible for the accelerated senescence phenotype and neuropathological changes in SAMP8 mice remain unclear. To explore the mechanism underlying the neuropathological changes and memory loss in SAMP8 mice, the expression of PAI-1 protein and cell cycle repressors p53 and p21 were assessed by Westerns. The results show that PAI-1 protein is significantly increased, which is correlated with the increases in p53 and p21 in the hippocampus/cortex of 12-month-old SAMP8 mice, compared to age-matched WT mice (Fig. 1A, B). Pearson Product Moment Correlation studies indicate that the amount of PAI-1 protein is closely correlated with the amounts of p53 (R=0.930, p<0.0001) and p21 (R=0.934, p<0.0001) (Fig. 1C). We also found that the increase of PAI-1 in the hippocampus/cortex of SAMP8 mice is age-dependent and so are the increases of p53 and p21 proteins (Fig. 1D-F). Immunofluorescence staining further show that the numbers of senescent astrocytes, which are stained positively with antibodies to macroH2A1a1 (a cell senescence marker) and GFAP (an astrocyte marker), are significantly increased with increased age in the hippocampus (Fig. 1G, H) and cortex of SAMP8 mice (Fig. 1G, I). Together, our data suggest that increased PAI-1 may underlie brain cell senescence, including astrocyte senescence, in SAMP8 mice.

**Figure 2.**
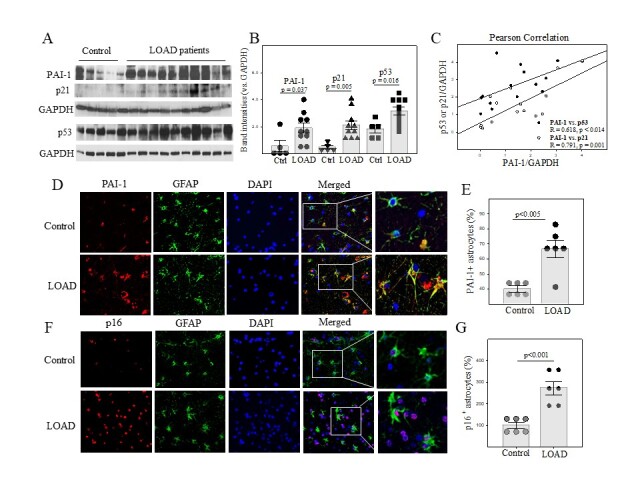
PAI-1 protein level is increased in the prefrontal cortex of LOAD patients. (A, B) Western analyses and quantifications of PAI-1, p53, and p21 proteins in the prefrontal cortex of LOAD patients and age-matched healthy controls. The band intensities presented were normalized by GAPDH (n=5-10). (C) Pearson correlation analysis of PAI-1 protein level and p53 protein/p21 protein level (n=15). (D, E) Double immunostaining and quantification of PAI-1 positive astrocytes in cortex slides from LOAD patients and control subjects (n=5-6). The results were expressed as percentages of controls. (F, G) Double immunostaining and quantification of p16 positive astrocytes in cortex slides from LOAD patients and control subjects (n=5-6). The results were expressed as percentages of controls.

### PAI-1 protein level is increased in correlation with increased expression of cell cycle repressors p53 and p21 in the prefrontal cortex of LOAD patients

Senescent cells, including astrocytes, microglia, endothelial cells, and neurons have been detected in the brain of AD patients. To define the role of PAI-1 in brain cell senescence in AD, we assessed the expression of PAI-1 as well as cell cycle repressor p53 in the dorsolateral prefrontal cortex of LOAD patients and age-matched healthy controls by Westerns. The results show that PAI-1 protein level is significantly increased in the dorsolateral prefrontal cortex of LOAD patients, relative to that in age-matched healthy controls (Fig. 2A, B). This is associated with increases in the expression of p53 and p21 (Fig. 2A, B). Pearson correlation studies show that the amounts of PAI-1 proteins in individual subjects are positively correlated with the amounts of p53 proteins (Correlation Coefficient 0.618, p<0.0141) and p21 protein (Correlation Coefficient 0.745, p<0.001) (Fig. 2C). Double immunostaining further showed that the numbers of PAI-1 positive astrocytes (Fig. 2D, E) and p16 positive astrocytes (Fig. 2F, G) are significantly higher in the dorsolateral prefrontal cortex of LOAD patients compared to that in age-matched healthy controls. These data suggest that more cells, including astrocytes, in LOAD patients compared to healthy controls, undergo senescence and that the increased PAI-1 expression may play a role in brain cell senescence in LOAD.

**Figure 3.**
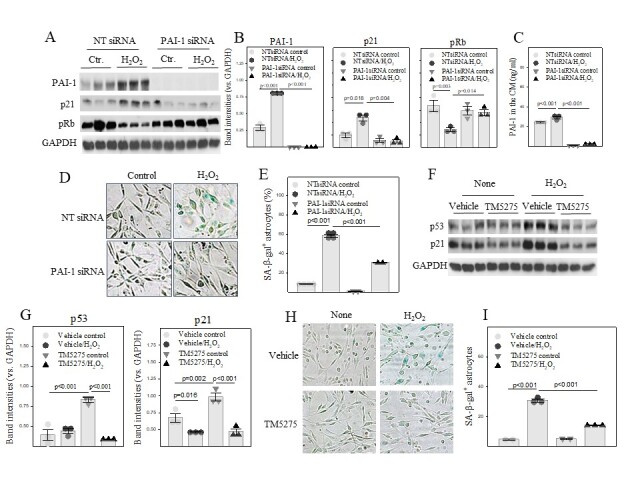
Silencing or inhibition of PAI-1 attenuates H_2_O_2_-induced U87 cell senescence. (A, B) Western analyses and quantification of PAI-1, p21, and phosphorylated Rb (pRb) proteins in U87 cells. The band intensities presented were normalized by GAPDH. C) ELISA of PAI-1 protein in the culture medium. (D, E) X-gal staining and quantification of SA-β-gal positive U87 cells. The results were expressed as percentages of total cells. (F, G) Western analyses and quantifications of p53 and p21 proteins in U87 cells. The band intensities presented were normalized by GAPDH. (H, I) X-gal staining of SA-β-gal positive U87 cells (n=3). The results were expressed as percentages of total cells.

### PAI-1 mediates H_2_O_2_-induced senescence in human astrocyte cell line U87 cells

To define the role of increased PAI-1 in astrocyte senescence, U87 cells, a human astrocyte cell line derived from malignant gliomas, were transfected with PAI-1 siRNA or non-targeted siRNA (NT-siRNA) and then treated with 200 μΜ H_2_O_2_. The results show that treatment of U87 cells with H_2_O_2_ increased PAI-1 protein levels in the cells and in the medium (Fig. 3A-C). H_2_O_2_ treatment also induced p21 and suppressed phosphorylation of retinoblastoma (pRb) (Fig. 3A, B), which was associated with an increase in the activity of senescence associated beta galactoses (SA-β-gal) (Fig. 3D, E), indicating that H_2_O_2_ induces U87 cell senescence. Silencing PAI-1, on the other hand, abrogated H_2_O_2_-induced p21 expression and Rb dephosphorylation (Fig. 3A-B). This was associated with a dramatic reduction of SA-β-gal activity (Fig. 3D, E), suggesting that increased PAI-1 mediate H_2_O_2_-induced U87 cell senescence. Moreover, we show that treatment with TM5275, a small molecule PAI-1 inhibitor, almost completely blocked H_2_O_2_-induced p53 and p21 expression (Fig. 3F, G) as well as SA-β-gal activity (Fig. 3H, I) in U87 cells. The data further support the notion that increased PAI-1 mediates H_2_O_2_-induced U87 cell senescence.

### PAI-1 mediates H_2_O_2_-induced senescence in primary human and mouse astrocytes

**Figure 4.**
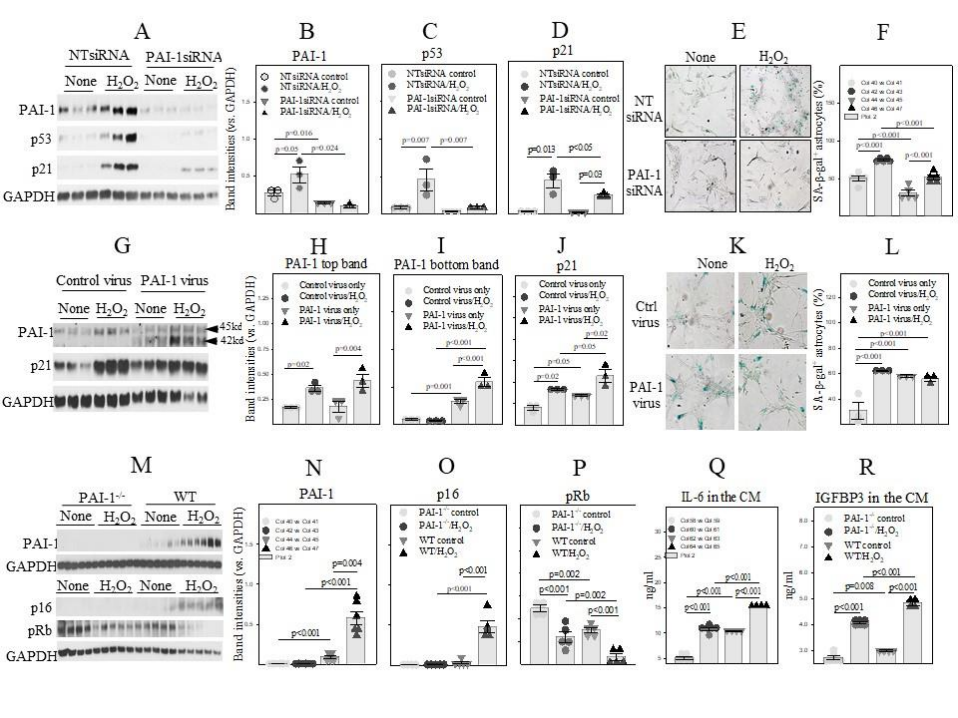
PAI-1 mediates H_2_O_2_-induced senescence in primary human and mouse astrocytes. (A-D) Western analyses and quantification of PAI-1, p53, and p21 in primary human astrocytes transfected with PAI-1siRNA/NTsiRNA and then treated with 200 μM H_2_O_2_/saline. The results were normalized by GAPDH (n=3). (E, F) X-gal staining and quantification of SA-β-gal positive astrocytes (n=5). The results were expressed as percentages of total cells. (G, H) X-gal staining of SA-β-gal positive astrocytes (n=3). The results were expressed as percentage of total cells. (I-L) Western analysis and quantifications of PAI-1 and p21 proteins. The band intensities presented were normalized by GAPDH. (M-P) Western analyses and quantifications of PAI-1, p16, and pRb proteins in primary astrocytes isolated from wild type or PAI-1^-/-^ mice treated with H_2_O_2_ or saline. The band intensities presented were normalized by GAPDH (n=4-5). (Q, R) ELISAs of IL-6 and IGFBP3 proteins in the culture medium (n=4-6).

To further confirm involvement of PAI-1 in astrocyte senescence, primary human astrocytes, passages 3-5, were treated with 200 μM H_2_O_2_ or transfected with PAI-1siRNA/NTsiRNA and then treated with H_2_O_2_. The results show that treatment with H_2_O_2_ induced PAI-1, p53, and p21 (Fig. 4A-D) and increased the activity of SA-β-gal (Fig. 4E, F). Silencing PAI-1, on the other hand, significantly reduced H_2_O_2_-induced p53 and p21 expression as well as SA-β-gal activity (Fig. 4A-F), suggesting that H_2_O_2_ induces primary human astrocyte senescence through a PAI-1 dependent mechanism. We also show that transduction of astrocytes with lentivirus expressing wild type of PAI-1 increased the amount of matured form of PAI-1 protein (MW 42kd), although it had no significant effect on the expression of the precursor form (MW 45kd) (Fig. 4G-I). H_2_O_2_ treatment, on the other hand, increased the amounts of both precursor form and matured form of PAI-1 proteins (Fig. 4G-I). Importantly, overexpression of PAI-1 alone increased p21 protein as well as SA-β-gal activity and enhanced H_2_O_2_-induced p21 expression, although it had no further effect on H_2_O_2_-induced SA-β-gal activity (Fig. 4G-L). Moreover, we show that ablation of PAI-1 in astrocytes (isolated from PAI-1^-/-^ mice) completely abolished H_2_O_2_-induced p16 expression and significantly attenuated H_2_O_2_-induced pRb dephosphorylation (Fig. 4M-P) as well as IL-6 and IGFBP3 secretion (Fig. 4Q, R). Together, our data suggest that PAI-1 mediates H_2_O_2_-induced senescence in primary human and mouse astrocytes.

**Figure 5.**
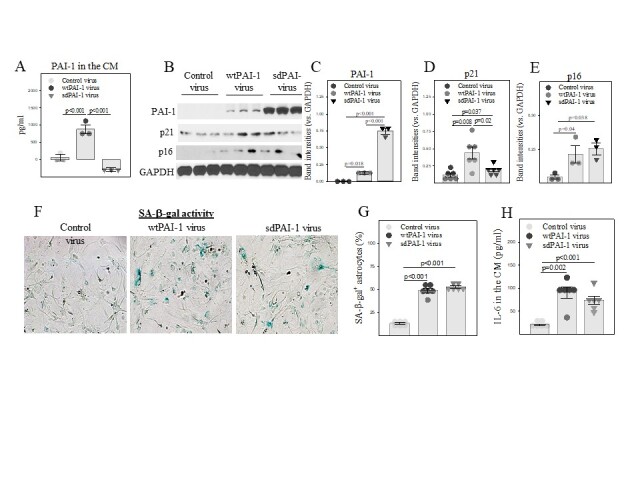
Overexpression of wild type or secretion deficient PAI-1 induces senescence in primary mouse astrocytes. (A) ELISA of PAI-1 protein in culture medium (CM). (B-E) Western analyses and quantification of PAI-1, p21, and p16 proteins in wtPAI-1, sdPAI-1, and control lentivirus-transduced primary PAI-1^-/-^ mouse astrocytes. The band intensities presented were normalized by GAPDH (n=3-6). (F, G) X-gal staining and quantification of SA-β-gal positive cells, the results expressed as percentages of total cells. (H) ELISA of IL-6 in the CM (n=6).

### Overexpression of PAI-1 alone, intracellularly, or extracellularly, induces senescence in primary mouse astrocytes

PAI-1, a secreted protein, is believed to function extracellularly and is an important component of senescence-associated secretory phenotype (SASP). To understand the molecular mechanism whereby PAI-1 promotes astrocyte senescence lentiviruses expressing wild type PAI-1 (wtPAI-1) and secretion deficient PAI-1 (sdPAI-1) were constructed in this lab. Primary astrocytes isolated from the cortex of PAI-1 deficient (PAI-1^-/-^) neonatal mice were infected with lentiviruses that express wtPAI-1, sdPAI-1, or virus vector only. The results show that wtPAI-1 is predominately present in the CM (extracellular), whereas sdPAI-1 appears only in the cell lysate (intracellular) (Fig. 5A-C), confirming that we have successfully constructed wtPAI-1 and sdPAI-1 lentiviruses. Importantly, we show, for the first time, that both wtPAI-1, which is presented mainly in the conditional medium (extracellularly), and sdPAI-1, which is only present in the cell lysates (intracellularly) (Fig. 5A-C), increased p21 and p16 expression (Fig. 5B, D, E) and SA-β-gal activity (Fig. 5F, G) and IL-6 secretion (Fig. 5H). These results suggest that overexpression of PAI-1 alone, intracellularly or extracellularly, can induce cell senescence.

### Astrocyte SASP promotes apoptotic responses in neurons and SASP PAI-1 mediates part of the effects

**Figure 6.**
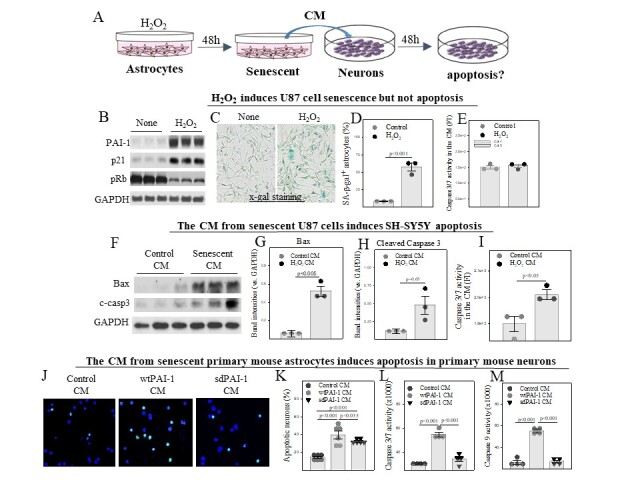
Astrocyte SASP promotes apoptotic responses in neurons. (A) Flow diagram showing the major steps of the experiments. (B-E) U87 cells were treated with 200 μM H_2_O_2_ for 48 hrs. (B) Western analyses of PAI-1, p21, and phosphorylated Rb proteins. (C, D) X-gal staining and quantification of SA-β-gal positive U87 cells (n=3). The results were expressed as percentages of total cells. E) Caspase activities in the conditional medium (CM) of U87 cells (n=3). F-I) SH-SY5Y cells were cultured with the CM from senescent U87 cells for 48 hrs. (F-H) Western analyses and quantifications of Bax and cleaved caspase3 proteins. The band intensities presented were normalized by GAPDH (n=3). (I) Caspase 3/7 activity in the culture medium of SH-SY5Y (n=3). (J-M) Primary mouse neurons were cultured with the CM from primary PAI-1^-/-^ mouse astrocytes transduced with wtPAI-1, sdPAI-1, or control lentiviruses as described in Fig 4 for 48 hours. (J, K) TUNEL staining of apoptotic neurons (n=6). The results were expressed as percentages of total cell number. (L) ELISA of caspase 3/7 and (M) ELISA of caspase 9 activities in the culture medium of primary neuron (n=4).

To explore the potential mechanisms by which senescent astrocytes contribute to AD neuropathology, SH-SY5Y cells, a neuroblastoma cell line, were cultured with the conditional medium (CM) from senescent U87 cells for 48 hours (Fig. 6A Flow chart). The results show that treatment with H_2_O_2_ induced U87 senescence (Fig. 6B-D) but had no significant effect on the activity of the caspase 3/7 (Fig. 6E), suggesting that H_2_O_2_ induces U87 senescence but not apoptosis. Importantly, we show that treatment with the CM (no H_2_O_2_ present, data not shown) from senescent U87 cells increased the expression of Bax and cleaved caspase 3 (Fig. 6F-H) as well as the activity of caspas3/7 (Fig. 6I) in SH-SY5Y cells, suggesting that U87 SASP promotes SH-SY5Y cell apoptosis.

To further investigate the role of SASP PAI-1 in astrocyte SASP-induced neuronal apoptosis, primary mouse cortical neurons were cultured with the CM from primary mouse astrocytes, which were isolated from PAI-1 deficient mice and transduced with control lentivirus, lentivirus expressing wild type PAI-1 (wtPAI-1) or secretion deficient PAI-1 (sdPAI-1). The CM did not contain viruses as the viruses were removed after overnight infection and the cells were cultured with viruses-free medium for 48 hours before the CM was collected. The results show that treatment of neurons with the CM from wtPAI-1 virus-infected PAI-1^-/-^ astrocytes led to significant increases in the number of TUNEL positive cells (Fig. 6J, K) as well as the activities of caspas3/7 and caspase 9 (Fig. 6L, M), compared to neurons treated with the CM from virus vector-transduced PAI-1^-/-^ astrocytes. Neurons treated with the CM from sdPAI-1 virus-infected PAI-1^-/-^ astrocytes also showed a significant increase in the number of TUNEL positive cells, although the increase was not as dramatic as wtPAI-1 CM treated neurons (Fig. 6J, K). Treatment of mouse neurons with the CM from sdPAI-1 virus-infected PAI-1^-/-^ astrocytes, however, did not significantly increase the activity of the caspas3/7 or caspase 9, although treatment of neurons treated with the CM from wtPAI-1-infected PAI-1^-/-^ astrocyte did (Fig. 6L, M). As both wtPAI-1 and sdPAI-1 induced astrocyte senescence (Fig. 5) and as no PAI-1 was present in the CM of sdPAI-1 virus-infected PAI-1^-/-^ astrocytes (Fig. 5A), our results indicate that SASP PAI-1 plays a role in astrocyte SASP-induced neuron apoptosis.

## DISCUSSION

**Figure 7.**
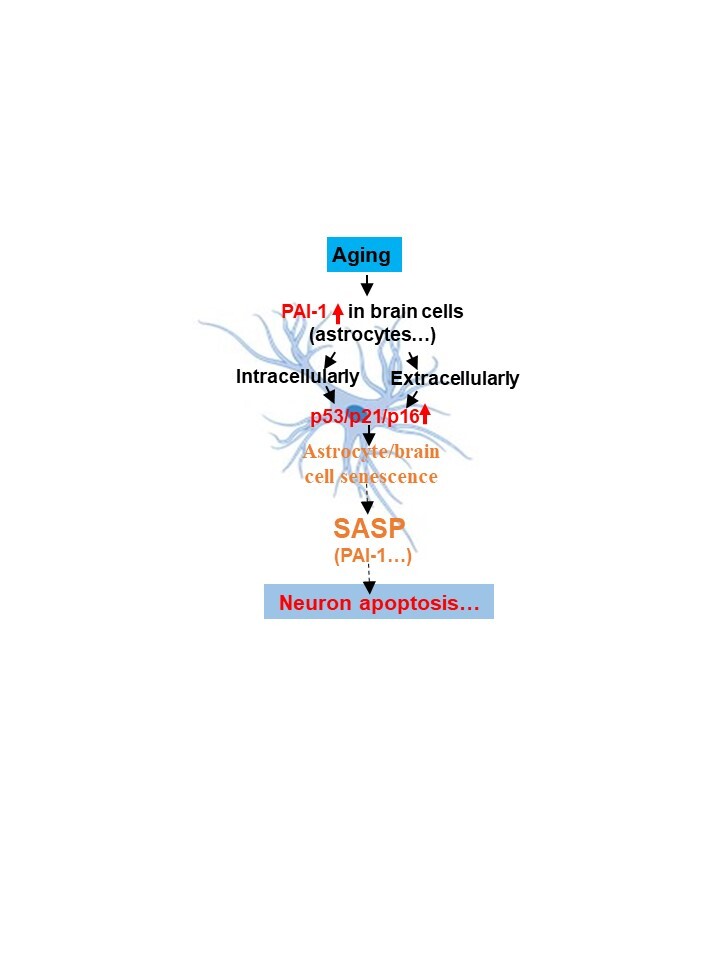
Hypothetic mechanism by which PAI-1 promotes brain cell senescence and neuron apoptosis during aging and in LOAD. PAI-1 expression increases with age and in LOAD brain. Increased PAI-1, intracellular or extracellular, leads to increases in the expression of cell cycle repressors p53, p21, and/or p16 as well as senescence in brain cells, including astrocytes. Senescent astrocytes in turn secrete pathologically active molecules, including PAI-1, which induces neuron apoptosis.

It has been well documented that the number of senescent cells increases with age and in aging-related diseases, including AD [1, 3-8, 38-40]. The mechanisms underlying cellular senescence during aging and in aging-related diseases, however, remain poorly understood. In this study, we show, for the first time, that the expression of PAI-1, a serine protease inhibitor that plays a central role in hemostasis, is significantly increased in the hippocampus/cortex and astrocytes in SAMP8 mice, an accelerated murine aging model, and in LOAD patients. This is correlated with the increases in the expression of cell cycle repressors p53, p21, and/or p16. Using primary human and mouse astrocytes, we further show that overexpression of PAI-1 alone intracellularly or extracellularly induced astrocyte senescence, whereas silencing or deletion of PAI-1 attenuated H_2_O_2_-induced p53/p21/p16 expression as well as astrocyte senescence. Moreover, we show that treatment of neurons with the conditional medium (CM) from senescent astrocytes induced neuron apoptosis whereas PAI-1 deficient CM had significantly less effect on neurons. As PAI-1 expression increases with age [9, 41] and in AD [11-13], our results suggest that increased PAI-1 may underline brain cell senescence and neuropathology during aging and in LOAD (Fig. 7).

Increased PAI-1 has been used as a marker of cell senescence and has been shown to mediate cell senescence in vitro and in vivo [34, 42-47]. Eren et al. showed that PAI-1 expression was increased in *Klotho* mice, another accelerated murine aging model [45]. Deletion of the PAI-1 gene in *Klotho* mice by crossing these mice with PAI-1 knockout mice reduced plasma levels of insulin-like growth factor binding protein 3 (IGFBP3) and IL-6, two of cell senescence markers and mediators [48]. Using a secretome proteomics approach, Elzi et al. identified IGFBP3 as a secreted mediator of breast cancer senescence upon chemotherapeutic drug treatment [43]. They further showed that the senescence-inducing activity of IGFBP3 was inhibited by tPA, whereas PAI-1 stabilized IGFBP3 by inhibiting tPA-mediated proteolysis of IGFBP3 [43]. Together, these data suggest that PAI-1 may mediate stress-induced cell senescence by increasing IGFBP3. Omer et al. reported, on the other hand, that sequestration of PAI-1 in stress granules (SGs) led to the translocation of cyclin D1 to nucleus and RB phosphorylation as well as suppression of senescence in two human diploid fibroblasts [46], suggesting that increased PAI-1 may promote cell senescence through modulating cyclin-pRb cell cycle repression pathway. In a previous study, we showed that specific ablation of PAI-1 in alveolar type II (ATII) cells in mice attenuated bleomycin-induced ATII cell senescence and lung fibrosis [34]. Using pharmacological and genetic approaches, we further showed that PAI-1 induced ATII cell senescence through activating p53-p21-pRb cell cycle repression pathways [34]. In contrast, we showed, in a different study, that PAI-1 mediated TGF-β1-induced ATII cell senescence through inducing p16, not p53 [47]. In this study, we further show that H_2_O_2_ induced primary human and mouse astrocyte senescence through activating p53/p21/p16 cell cycle repressors. Although the underlying mechanism is still unknown, these data suggest that increased PAI-1 may promote brain cell senescence through increasing the expression of cell cycle repressors.

PAI-1 is a secreted protein and is believed to function mainly in extracellular space, although a few studies suggest that intracellular PAI-1 may also have important functions [18-20]. To define the role of intracellular PAI-1 in cell senescence, we constructed a secretion deficient PAI-1 (sdPAI-1) expression lentivirus by deletion of the nucleotides coding the secretion signal peptides. We found that transduction of PAI-1^-/-^ astrocytes with sdPAI-1 expression lentivirus alone induced astrocyte senescence (increased p16 expression and SA-β-gal activity), just like wild type PAI-1 (wtPAI-1) expression lentivirus did. These data suggest that an increase in the intracellular PAI-1 alone is sufficient to activate cell cycle repression pathway and induce senescence. Our data further support the notion that intracellular PAI-1 also has important functions, although the mechanism by which intracellular and extracellular PAI-1 activates cell cycle repression pathways remains to be further investigated.

Senescence accelerated murine prone 8 (SAMP8) mice are a naturally occurring mouse line displaying a phenotype of accelerated aging [49, 50]. The lifespan of SAMP8 mice is 40% shortened [21, 50] and these mice harbor many behavioral and histopathological signatures of AD, including Aβ deposition, tauopathy, and memory deficits [26, 31, 35-37]. Although the accelerated aging and neuropathophysiology phenotypes have been well characterized in SAMP8 mice, the gene(s) that is (are) responsible for the phenotypes remain unclear. In this study, we show for the first time that PAI-1 expression is significantly increased in the hippocampus/cortex of SAMP8 mice, associated with increased expression of cell cycle repressors p53 and p21, suggesting that increased PAI-1 may underlie the aging phenotype. This notion is supported by the observations from *Klotho* mice, another murine aging model [45]. Eren et al. reported that PAI-1 expression has increased in *Klotho* mice. Deletion of the PAI-1 gene reversed senescence phenotype, preserved organ structure and function, and prolonged the lifespan in *Klotho* mice [45]. Most interestingly, Khan et al. reported that people at Berne Amish community who carry heterozygous mutation (c.699_700upTA) in the PAI-1 gene with a loss of function of the protein have significantly longer leukocyte telomere length, lower fasting insulin levels, and lower prevalence of diabetes mellitus whereas the carriers of the null PAI-1 allele had a longer life span [51]. These data further support the critical role of PAI-1 in aging processes in humans. Nonetheless, whether increased PAI-1 is responsible for the accelerated aging phenotype and neuropathophysiology in SAMP8 mice and in human population remains to be further investigated.

Although emerging evidence indicates that cell senescence plays a critical role in the pathophysiology of aging and aging-related diseases, including AD, the underlying mechanism remains unclear. In a previous study, we showed that treatment of alveolar macrophages with the conditional medium (CM) from senescent type II alveolar epithelial (ATII) cells stimulated the expression of the genes associated with a pro-fibrotic phenotype in alveolar macrophages [47]. Deletion or inhibition of PAI-1 attenuated TGF-β1 induced ATII cell senescence, SASP, and the stimulatory effects of ATII cell SASP on macrophages [47]. These data support the notion that senescent cells secret biologically active molecules that adversely affect the survival or function of adjacent cells. Astrocytes, the most abundant cell type in the brain, are closely associated with neurons and are essential for neuron survival and function. In this study, we show, for the first time, that the CM from senescent astrocytes promotes apoptotic responses in neurons. Importantly, the CM containing no PAI-1 had a significantly reduced capacity to induce neuron apoptosis compared to PAI-1 contained CM, although secretion deficient PAI-1 induced similar levels of astrocyte senescence as wild type PAI-1. These data suggest that senescent astrocytes can promote neuron apoptosis by secreting pathogenically active molecules including PAI-1. More works are needed to understand how SASP PAI-1 promotes neuron apoptosis.

In summary, we show for the first time that PAI-1 expression is increased, in correlation with the increased expression of cell cycle repressors p53/p21/p16, in the hippocampus/cortex of SAMP8 mice and LOAD patients. In vitro studies further suggest that increased PAI-1 expression may underlie astrocyte senescence and that senescent astrocytes can promote neuron apoptosis by secreting pathologically active molecules, including PAI-1. More works are needed to elucidate the mechanism by which PAI-1 regulates the expression of cell cycle repressors in astrocytes and how SASP promotes neuron apoptosis. Whether increased PAI-1 is responsible for the aging phenotype in SAMP8 mice and in human being also warrants further investigation.
